# Karyotypic Profiling of Induced Pluripotent Stem Cells Derived from a Xeroderma Pigmentosum Group C Patient

**DOI:** 10.3390/cells14241985

**Published:** 2025-12-14

**Authors:** Almaqdad Alsalloum, Natalia Mingaleva, Ekaterina Gornostal, Zoia Antysheva, Peter Sparber, Mikhail Skoblov, Victoria Pozhitnova, Tatiana Belysheva, Aygun Levashova, Ekaterina Kuznetsova, Yulia Suvorova, Julia Krupinova, Viktor Bogdanov, Alexej Abyzov, Olga Mityaeva, Pavel Volchkov

**Affiliations:** 1 Federal Research Center for Innovator and Emerging Biomedical and Pharmaceutical Technologies, 125315 Moscow, Russia; alsallum_a@academpharm.ru (A.A.);; 2 Moscow Center for Advanced Studies, Kulakova Str. 20, 123592 Moscow, Russia; 3 Research Centre for Medical Genetics, 115522 Moscow, Russia; 4 N.N. Blokhin National Medical Research Center of Oncology, 127224 Moscow, Russia; 5 Limited Liability Company “Biotechnology Campus”, 117997 Moscow, Russia; 6 Moscow Clinical Scientific Center N.A. A.S. Loginov, 111123 Moscow, Russia; 7 Department of Quantitative Health Sciences, Mayo Clinic, Rochester, MN 55905, USA; 8 Department of Fundamental Medicine, Lomonosov Moscow State University, 119992 Moscow, Russia

**Keywords:** xeroderma pigmentosum group C (XP-C), DNA repair deficiency, induced pluripotent stem cells (iPSCs), chromosomal aberrations, G-banding

## Abstract

Xeroderma Pigmentosum group C (XP-C) is an autosomal recessive disorder caused by mutations in the *XPC* gene, leading to defective nucleotide excision repair. This defect leads to genomic instability and a profound cancer predisposition. To model this disease, we generated induced pluripotent stem cells (iPSCs) from an XP-C patient carrying a novel homozygous nonsense mutation in the *XPC* gene (c.1830C>A). The resulting iPSCs demonstrated typical pluripotent characteristics, including expression of key markers and trilineage differentiation capability. However, genomic assessment revealed progressive karyotypic instability during extended culture. While initial whole-genome sequencing detected no major chromosomal abnormalities, subsequent G-banding analysis identified acquired trisomy 12 in two lines (CL12 and CL27) and a derivative X chromosome in a third line (CL30). These abnormalities were absent in early-passage analyses, indicating that they were acquired and selected for during extended culture. The acquisition of a derivative X chromosome in CL30, alongside recurrent trisomy 12, represents a unique cytogenetic signature likely attributable to the underlying XPC defect. We hypothesize that the loss of GG-NER creates a permissive genomic environment, accelerating the accumulation of DNA damage and chromosomal missegregation under replicative stress. This temporal divergence in genomic integrity highlights how culture pressures drive chromosomal evolution in XP-C iPSCs independently of initial reprogramming. Our findings emphasize that XP-C iPSCs require continuous genomic surveillance and provide a model for investigating how DNA repair deficiencies interact with in vitro culture stress.

## 1. Introduction

Xeroderma pigmentosum (XP) is a rare genetic disorder characterized by profound hypersensitivity to ultraviolet (UV) radiation, a dramatically elevated risk of cutaneous malignancy, and frequent neurological manifestations. The condition is stratified into seven complementation groups (XP-A to XP-G) and a variant form (XP-V), each corresponding to a specific genetic defect in the nucleotide excision repair (NER) pathway [1,2]. Researchers have identified a novel, ninth XP complementation group, designated XP-J. The classification is based on a pediatric patient with a classic XP phenotype who was unassignable to any known group, with the disorder traced to biallelic mutations in the *GTF2H4* gene [3,4]. Notably, individuals with group C (XP-C), which arises from mutations in the *XPC* gene, exhibit a pronounced susceptibility to a spectrum of malignancies, including basal cell carcinoma, squamous cell carcinoma, melanoma, and sarcomatoid carcinoma. The XPC protein serves as a critical initiator in the global genome-NER (GG-NER) subpathway, where it is essential for the primary recognition of helical distortions in DNA. This function is vital for reducing the mutational burden and maintaining genomic integrity [1,2].

The clinical phenotype of XP is highly heterogeneous and correlates with the specific genetic complementation group. The spectrum of disease ranges from patients with acute cutaneous photosensitivity to those who develop numerous non-melanoma skin cancers (NMSCs) at an early age. A significant subset of patients also experiences progressive neurological degeneration, which can be accompanied by features of premature aging. Neurological manifestations in XP are most prevalent in the XP-A, XP-D, and XP-G complementation groups. These patients collectively account for an estimated 20–30% of all XP cases [5,6]. Patients with XP exhibit a significantly elevated risk of internal tumors, associated with both specific characteristic mutations and the accumulation of multiple mutations [7,8]. Furthermore, XP patients with neurological manifestations demonstrate more severe impairment in DNA repair following UV exposure compared to those without such disorders. This suggests a relationship between the balance of DNA damage and repair, and the initiation of tumors associated with UV exposure [9,10]. The susceptibility to neurological damage in XP varies significantly based on the specific causative mutation. Different allelic variants within a single XP gene produce proteins with a spectrum of residual activity. Crucially, the integrity of the transcription-coupled nucleotide excision repair (TC-NER) pathway—which rapidly repairs damage on the transcribed strand of active genes—serves as the primary determinant of neuroprotection. Severe, loss-of-function mutations like frameshifts that abolish TC-NER are strongly associated with progressive neurological degeneration. In contrast, hypomorphic mutations, often missense in nature, can retain partial TC-NER activity. This residual capacity is sufficient to prevent catastrophic neuronal loss, resulting in a phenotype where patients present with the dermatological signs of XP but are either neurologically spared or experience a markedly attenuated and delayed neurological course [11].

The molecular basis of XP is defined by defective DNA repair, which leads to the accumulation of mutagenic UV-induced lesions. In most complementation groups like XP-C and XP-E, this defect lies in GG-NER, resulting in a genome-wide burden of unrepaired damage that stalls DNA replication. To ensure replication proceeds, cells employ error-prone translesion synthesis (TLS) polymerases, such as DNA polymerase η, to bypass the lesions. This process promotes cell survival at the cost of heightened mutagenesis, directly accounting for the profound cancer predisposition in GG-NER-deficient patients. Conversely, the XP variant (XP-V) form results from mutations in the *POLH* gene, which inactivate polymerase η itself. This ablation of efficient TLS prevents lesion bypass, leading to replication fork collapse. This crisis forces cells to utilize alternative, highly mutagenic DNA repair pathways, which ultimately generate a similarly elevated mutational burden and cancer susceptibility [12,13,14]. Pathogenic variants in the *XPC* gene are the most prevalent, accounting for approximately 43% of XP cases. However, significant genetic heterogeneity exists within the XPC group itself, arising from diverse mutational sites across the gene. This heterogeneity complicates the establishment of clear genotype-phenotype correlations, making it challenging to definitively link specific mutation sites and locations with the resulting clinical symptoms [15].

A molecular investigation utilizing patient-derived fibroblasts with various *XPC* gene mutations established that full-length XPC protein was absent, despite detectable levels of XPC mRNA. This indicates that the mutant transcripts likely harbor premature termination codons (PTCs), which target them for degradation via the nonsense-mediated mRNA decay (NMD) pathway, thereby preventing the synthesis of truncated and potentially harmful protein products. These findings are consistent with the established disease mechanism, whereby biallelic pathogenic variants in the *XPC* gene lead to a loss of function, such as nonsense, frameshift, or deletion/duplication mutations [16]. However, patient-derived fibroblasts are insufficient for modeling the disease’s long-term progression. Induced pluripotent stem cells (iPSCs) overcome this limitation by providing a self-renewing, patient-specific model. This system is therefore crucial for directly linking the continuous absence of functional XPC to the chromosomal aberrations and impaired differentiation that define disease pathogenesis.

iPSC technology is widely recognized as a powerful tool for modeling disease pathology, especially in the context of human genetic diseases caused by impairments in DNA repair mechanisms [17,18]. This technology enables the development of in vitro models for drug screening and potential therapeutic interventions assessment. To date, there has been a scarcity of studies generating and applying iPSC-based models to elucidate the pathogenic mechanisms and progression of XP [19]. Previous studies suggested that somatic cells from patients with DNA repair disorders are difficult to reprogram, one study successfully generated iPSCs from XP patients without observing impaired reprogramming kinetics. The same research team demonstrated that these XP-derived iPSCs maintained the potential to differentiate into all three germ layers and preserved chromosomal stability. Consequently, the study concluded that XP-specific iPSCs represent a promising platform for investigating the disease mechanism, particularly the relationship between XP disorders and UV-induced malignant transformation [19].

The cultivation of iPSCs can be challenged by genomic instability, frequently attributed to the stresses of the reprogramming process itself. Investigations into chromosomal aberrations in both human embryonic stem cells (ESCs) and iPSCs have consistently identified trisomy 12 as the most recurrent anomaly. Research indicates that a fraction of the genetic variations detected in iPSCs are pre-existing in the source somatic cells and become fixed during the clonal expansion of reprogramming. Furthermore, prolonged in vitro culture can independently compromise chromosomal stability. Mutations arising from culture conditions are typically stochastic and are expected to exhibit lower allele frequencies within the cell population. Ultimately, the efficiency and safety of reprogramming—and by extension, the genomic stability of the resulting iPSCs—are influenced by multiple variables. These include the choice of starting cell source, the delivery method for the reprogramming factors, and the specific factors used [20].

In this study, we generated iPSCs from a patient with Xeroderma Pigmentosum group C (XPC). Contrary to previous reports of stable XP-specific lines, we observed significant karyotypic instability across multiple patient-derived lines. We hypothesize that the specific genetic context of our XPC patient—harboring a complete loss-of-function mutation—confers a unique susceptibility to replication or mitotic stress, predisposing the cells to genomic instability in vitro. Initial whole-genome sequencing (WGS) did not reveal major structural variants. Therefore, we are undertaking a detailed, cross passages characterization of genomic integrity in these XP-C iPSCs to elucidate the dynamics of this instability and test our hypothesis.

## 2. Materials and Methods

### 2.1. Clinical Subject

A 15-year-old female proband was evaluated at the N.N. Blokhin National Medical Research Center of Oncology, Moscow, Russia. Clinical geneticists obtained a detailed medical history, performed a comprehensive dermatological evaluation, and conducted confirmatory genetic testing to establish the diagnosis.

### 2.2. PBMC Isolation and Reprogramming

Peripheral blood mononuclear cells (PBMCs) were isolated from the patient using Ficoll™ (STEMCELL Technologies, Vancouver, BC, Canada) density gradient centrifugation. The isolated PBMCs were cultured for four days in StemPro™-34 SFM medium (ThermoFisher, Waltham, MA, USA), supplemented with 100 ng/mL each of SCF and FLT-3, 20 ng/mL each of IL-3 and IL-6 (all from ThermoFisher), in presence of 1× Antibiotic/Antimycotic Solution (Capricorn, Ebsdorfergrund, Germany).

On the day of transduction, cells were harvested and transduced with the CytoTune^®^-iPS 2.0 Sendai Virus reprogramming kit (Life Technologies, Invitrogen, Carlsbad, CA, USA) at a multiplicity of infection (MOI) of 5:5:3 for the KOS, hc-Myc, and hKlf4 vectors, respectively. The viral supernatant was removed 24 h post-transduction, and the cells were cultured for an additional two days in cytokine-free StemPro™-34 SFM. During this period, a morphological shift was observed, characterized by cell enlargement, surface attachment, and the initial formation of colonies. On the third day post-transduction, 2.0–3.0 × 10^5^ cells were plated onto a 12-well plate coated with Matrigel (Corning, NY, USA). The following day, the medium was gradually replaced with mTeSR™1 (STEMCELL Technologies). Distinct induced pluripotent stem cell (iPSC) colonies emerged by day 10 and were manually isolated for expansion.

### 2.3. iPSC Culture and Maintenance

For routine maintenance, the established iPSC cultures were passaged at 70–90% confluency using TrypLE™ Express Enzyme (ThermoFisher). The dissociated cells were incubated overnight in mTeSR™1 medium supplemented with 10 µM thiazovivin (STEMCELL Technologies) in a chamber at 37 °C with 5% CO_2_ and 5% O_2_. The following day, the medium was completely replaced with fresh mTeSR™1 containing 1× Antibiotic/Antimycotic, without the ROCK inhibitor (thiazovivin).

### 2.4. Pluripotency Characterization

#### 2.4.1. Immunocytochemistry (ICC)

Immunofluorescence staining was performed to assess the expression of key pluripotency markers in the iPSC colonies. The analysis was conducted at passage 16 when cultures reached approximately 50% confluence to ensure optimal marker expression and minimize spontaneous differentiation. The staining procedure was carried out as follows. First, the culture medium was aspirated, and the cells were washed gently three times with phosphate-buffered saline (PBS) (Paneco, Moscow, Russia) to remove residual media and debris. The cells were then fixed for 15 min at room temperature using a 4% solution of paraformaldehyde (PFA) (Sigma-Aldrich, Burlington, MA, USA) in PBS. Following fixation, the PFA was quenched and removed with three subsequent PBS washes.

To permit intracellular antibody access, the fixed cells were permeabilized by incubating with 0.2% Triton X-100 (AppliChem, Darmstadt, Germany) in PBS for 10 min at room temperature. Non-specific binding sites were subsequently blocked by treating the cells with a blocking solution of 2.5% bovine serum albumin (BSA) (Paneco) in PBS for 60 min at room temperature. The cells were then incubated with primary antibodies for two hours at room temperature. The primary antibodies, targeting the pluripotency markers OCT4, SOX2, TRA-1-60, and SSEA-4, were diluted to their manufacturer-specified working concentrations in a solution of 1% BSA in PBS. Following incubation, unbound primary antibodies were removed by washing the cells three times for five minutes each with a PBS solution containing 0.1% Tween 20 (Bio-Rad, Hercules, CA, USA).

Next, the corresponding fluorophore-conjugated secondary antibodies, diluted in 1% BSA in PBS, were applied to the cells and incubated for 30 min at room temperature in the dark to prevent photobleaching. After incubation, the cells were washed twice with PBS for five minutes per wash to remove excess secondary antibodies. To visualize cell nuclei, the samples were counterstained with 0.1 µg/mL DAPI (Lumiprobe, Moscow, Russia) in PBS for 5 min, followed by a final two brief washes with PBS. Imaging was performed using an EVOS M5000 Imaging System (ThermoFisher Scientific, Waltham, MA, USA). A complete list of antibodies, including catalog numbers, dilutions, and hosts, is provided in Supplementary Table S1.

#### 2.4.2. Flow Cytometry (FC)

For the quantitative analysis of pluripotency marker expression, iPSC colonies at passage 16 were harvested and dissociated into a single-cell suspension using TrypLE™ Express Enzyme. The cells were washed with PBS and prepared for flow cytometry. Two distinct staining protocols were employed based on the subcellular localization of the target antigens. For the intracellular transcription factors OCT4 and SOX2, cells were fixed with 1% PFA, permeabilized with 0.2% Triton X-100, and blocked with 2.5% BSA. For the surface markers TRA-1-60 and SSEA-4, cells were blocked with 2.5% BSA without prior fixation or permeabilization. Following blocking, all samples were incubated with the appropriate primary antibodies for 30 min at room temperature, followed by fluorochrome-conjugated secondary antibodies for 20 min. All antibodies were diluted in 1% BSA. Cells were then counterstained with 0.1 µg/mL DAPI to label nuclei. Samples were analyzed using a CytoFLEX flow cytometer (Beckman Coulter, Brea, CA, USA), and data from at least 10,000 events were collected for subsequent analysis.

#### 2.4.3. In Trilineage Differentiation

To induce spontaneous differentiation via embryonic body (EB) formation, iPSC colonies at passage 15 were harvested at 80% confluency using TrypLE™ Express Enzyme. A total of 8 × 10^5^ cells per well were plated in 3 mL of mTeSR™ medium into ultra-low attachment 6-well plates (Corning), supplemented with 1 µM Thiazovivin to enhance cell survival. The EBs were maintained in suspension culture for eight days. On day 3 and day 6, two-thirds of the medium was carefully replaced with fresh mTeSR™. On day 8, the resulting EBs were individually transferred to a 0.1% gelatin (Paneco)-coated 96-well plate and switched to a differentiation medium consisting of DMEM/F-12 (ThermoFisher) supplemented with 10% fetal bovine serum (FBS) (ThermoFisher) and 1× GlutaMAX (ThermoFisher). This medium was replaced every three days. After two weeks of adherent culture, the differentiated cells were fixed and assessed for the presence of derivatives of the three germ layers (ectoderm, mesoderm, and endoderm) by immunocytochemistry, following the standard protocol described previously. Antibodies specific to canonical markers for each germ layer were used for this analysis. See Supplementary Table S1 for details.

### 2.5. Quality Control Assays

#### 2.5.1. Mycoplasma Testing

The cell cultures were routinely screened for mycoplasma contamination using the MycoReport detection kit (Evrogen, Moscow, Russia), following the manufacturer’s protocol.

#### 2.5.2. Sendai Virus Clearance Assay

To assess reprogramming efficiency, RNA was extracted from cells at day 3 of the reprogramming process and from stabilized iPSC lines using the RNeasy kit (Qiagen, Hilden, Germany). cDNA was synthesized and subsequently amplified by RT-PCR using primers recommended by the CytoTune^®^-iPS 2.0 Reprogramming Kit manufacture protocol (Life Technologies, Invitrogen). Primer sequences and their expected product sizes are provided in Supplementary Table S2.

#### 2.5.3. STR Analysis

Cell line authentication was conducted to ensure the genetic fidelity of the reprogrammed iPSCs. Genomic DNA was extracted from iPSCs at passage 11 using the ExtractDNA Blood Kit (Evrogen). The genetic match between the established iPSC line and the original donor PBMCs was obtained by analyzing 20 short tandem repeat (STR) loci with the COrDIS Plus STR Amplification Kit (Gordiz Service, Moscow, Russia).

#### 2.5.4. Mutation Verification

Following the manufacturer’s protocol for the MagPure Universal DNA Kit (Magenб Guangzhou, China), genomic DNA was extracted from cells at passage 17. The target regions were amplified by PCR, and the amplicons were verified using Sanger sequencing. Primer sequence details are provided in Supplementary Table S2.

### 2.6. Karyotype Analysis

iPSCs at passage 15 and approximately 70% confluency were subjected to karyotypic analysis. To prepare metaphase chromosomes, cells were mitotically synchronized for 1 h using 0.1 μg/mL demecolcine (Sigma-Aldrich). The synchronized cells were harvested via trypsinization (Paneco) to ensure optimal colony dissociation, followed by hypotonic treatment with prewarmed 0.075 M KCl for 13 min at 37 °C. Cell fixation was performed in three changes of an ice-cold 3:1 methanol/glacial acetic acid mixture (Chimmed, Moscow, Russia). The fixed cell suspension was dropped onto microscope slides (Menzel Glaser, Braunschweig, Germany) and air-dried. For chromosome banding, slides were stained with Vectashield antifade mounting medium containing DAPI (Vector Laboratories, Newark, CA, USA) for nuclear visualization and premixed with 0.3 mg/mL actinomycin D (Serva, Heidelberg, Germany) to enhance band contrast.

Metaphase images were captured using an Axio Imager 2 microscope (Zeiss, Oberkochen, Germany) equipped with a camera. The images were digitally inverted and processed using the “Q-banding” mode of the Case Data Manager 6.0 software (Applied Spectral Imaging, Carlsbad, CA, USA). Karyotypes were analyzed according to the International System for Human Cytogenomic Nomenclature (ISCN 2020). A minimum of 15–20 high-quality metaphase spreads were examined for chromosomal abnormalities.

### 2.7. Whole Genome Sequencing (WGS) and Analysis

Genomic DNA was isolated from all samples at passage 8 utilizing magnetic bead-based purification with the MGIEasy Magnetic Beads Blood Genomic DNA Extraction Kit (MGI, Shenzhen, China). Sequencing libraries were constructed using the MGIEasy FS PCR-Free DNA Library Prep Set (BGI, Shenzhen, China). Whole-genome sequencing was subsequently performed on a DNBSEQ-T7 platform in paired-end mode (2 × 150 bp), achieving a mean on-target coverage of 30× across the sample set. Bioinformatic processing of the raw sequencing data was conducted as follows. Adapter sequences and low-quality bases were trimmed from the raw reads using fastp (v0.23.2). The resulting high-quality reads were aligned to the UCSC GRCh38/hg38 human reference genome using BWA-MEM2 (v2.2.1) [21,22]. PCR duplicates were marked and removed, and mate-pair information was corrected using samtools (v1.21). The depth of coverage was quantified with mosdepth (v0.3.3), which confirmed a final mean coverage of 34.2×. For the detection of copy number variations (CNVs), we employed Control-FREEC (v11.6) [23]. The statistical significance of the identified genomic segments was assessed using a Wilcoxon rank-sum test, with segments yielding a *p*-value < 0.05 considered significant. Visualization of the resulting CNV segments was performed using the matplotlib (v3.10.3) and seaborn (v0.13.2) libraries in Python [24].

## 3. Results

### 3.1. Clinical Presentation of the XP-C Proband

The proband is a 15-year-old female who first presented for genetic evaluation at the age of 8, following the emergence of a persistent skin rash and xerosis in sun-exposed regions, with symptom onset noted in her second year of life. The most recent clinical examination revealed significant cutaneous manifestations. The skin in photo-exposed areas—including the face, neck, and upper and lower extremities—exhibited a coarse texture with a characteristic mottled hyperpigmentation. Early signs of degenerative cartilaginous changes were also apparent, manifesting as deformation and thinning of the nasal architecture and auricles, accompanied by the development of microstomia.

The dermatological presentation was consistent with classical poikiloderma, characterized by a triad of features: multiple hyperpigmented lentigines (ranging from 0.3 to 0.5 cm in diameter), numerous telangiectasias, and focal areas of dermal atrophy, predominantly localized to the zygomatic and infraorbital regions, as well as the dorsum and tip of the nose. A suspicious grayish papule, 0.4 cm in diameter, was observed on the nasal tip amidst prominent telangiectasias, raising clinical suspicion of basal cell carcinoma. Actinic cheilitis was evident, presenting as persistent inflammation, atrophy, xerosis, and scaling of the vermilion border. A separate, flesh-colored papule with a verrucous surface and irregular borders, measuring 0.5 cm, was identified on the left lower lip.

Based on this constellation of classic clinical findings, a diagnosis of xeroderma pigmentosum was established. Molecular genetic analysis via whole-genome sequencing identified a previously unreported homozygous nonsense variant in the *XPC* gene: NM_004628.4: c.1830C>A, resulting in a premature termination codon, p.(Tyr610*). In accordance with the American College of Medical Genetics and Genomics (ACMG) guidelines, this variant was classified as likely pathogenic (criteria: PVS1, PM2), thereby providing molecular confirmation of the clinical diagnosis.

### 3.2. Generation and Validation of XP-C iPSCs

**Figure 1:**
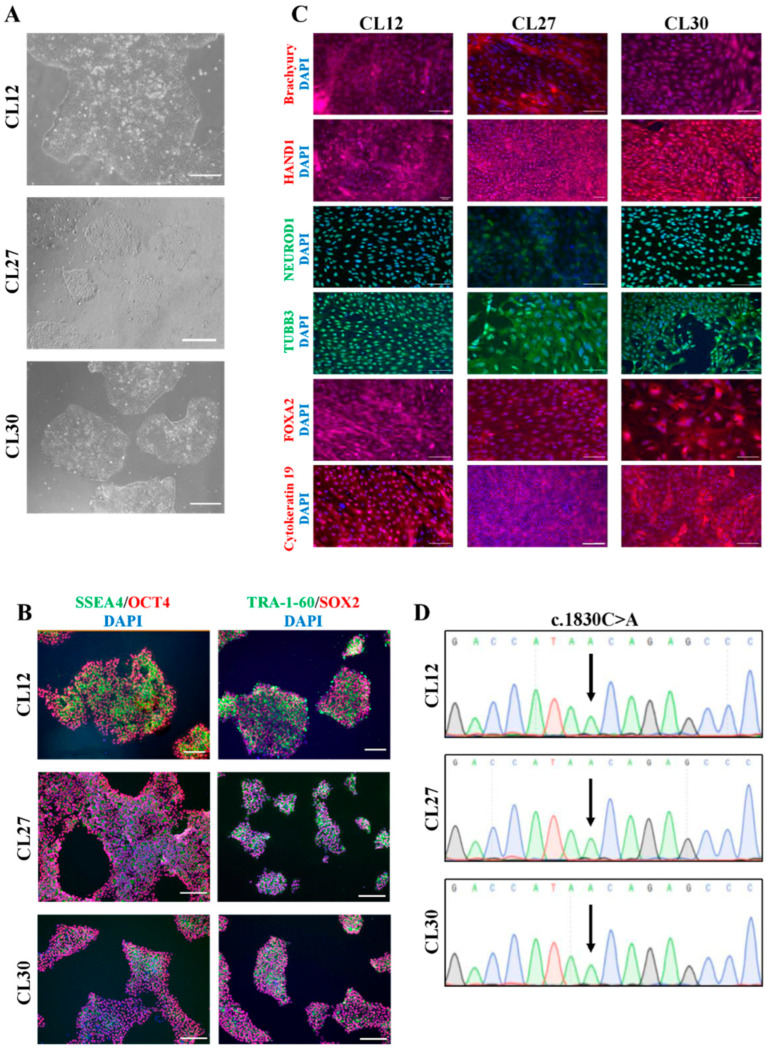
Characterization of the patient-derived iPSC lines with a homozygous *XPC* mutation. (**A**) Representative bright-field images showing the characteristic embryonic stem cell-like morphology of the established iPSC lines (CL12, CL27, CL30). (**B**) Immunofluorescence analysis of key pluripotency markers. Images show robust expression of the surface markers SSEA-4 and TRA-1-60, and the nuclear transcription factors OCT4 and SOX2. (**C**) Immunofluorescence analysis of embryonic bodies (EBs) derived from the iPSC lines for markers of the three germ layers: Endoderm (FOXA2, Cytokeratin 19), Mesoderm (Brachyury, HAND1), and Ectoderm (NeuroD1, TUBB3). Nuclei are counterstained with DAPI. Scale bars: 100 µm. (**D**) Sanger sequencing chromatograms confirming the homozygous c.1830C>A mutation (arrows) in the *XPC* gene for the three iPSC lines (CL12, CL27, CL30).

This study established three patient-derived iPSC lines carrying a homozygous mutation in the *XPC* gene, obtained from an individual diagnosed with XP. To investigate the effects of DNA repair gene mutations and the complexities of reprogramming somatic cells from patients with inherited DNA repair deficiencies, PBMCs from the patient were reprogrammed using a Sendai viral vector encoding pluripotency factors. Individual clonal lines were manually isolated and expanded separately, preventing cross-contamination. Multiple colonies emerged approximately two weeks post-transduction. Upon reaching sufficient size for passaging, these colonies exhibited a characteristic embryonic stem cell (ESC)-like morphology, featuring densely packed cells with high nucleus-to-cytoplasm ratios, prominent nucleoli, and indistinct cell borders (Figure 1A).

Several clonal lines (CL12, CL27, CL30) were selected for comprehensive characterization. Pluripotency was validated using both qualitative and quantitative methods. Immunocytochemical staining confirmed robust expression of key pluripotency markers, including the intracellular transcription factors OCT4 and SOX2, and the surface markers SSEA-4 and TRA-1-60 (Figure 1B). Flow cytometric analysis demonstrated that over 90% of cells in each line expressed these markers (Supplementary Figure S1). Furthermore, the iPSCs successfully formed embryonic bodies (EBs). Immunofluorescence analysis of these EBs confirmed their capacity to differentiate into all three germ layers, as evidenced by positive staining for endodermal (FOXA2, Cytokeratin 19), mesodermal (Brachyury, HAND1), and ectodermal (NeuroD1, TUBB3) markers (Figure 1C).

To confirm the retention of the mutation in vitro, Sanger sequencing of the generated iPSC lines was performed. This analysis verified the presence of the same novel homozygous *XPC* variant (c.1830C>A) (Figure 1D) that had been initially identified in the patient’s DNA via whole-genome sequencing. Short tandem repeat (STR) profiling confirmed the genetic identity of the iPSCs with the original patient PBMCs. Additional quality control measures, including RT-PCR, verified the absence of the reprogramming transgene and Sendai virus (Supplementary Figure S2), while PCR testing confirmed the cell line was free of mycoplasma contamination (Supplementary Figure S3). In conclusion, the iPSC lines were successfully established through reprogramming without observed technical difficulties, providing a validated model for future studies.

### 3.3. Karyotypic Evolution in XP-C iPSCs During In Vitro Culture

To ensure the genomic integrity of the iPSC line—a critical prerequisite for reliable downstream applications including in vitro differentiation modeling and therapeutic development—a cytogenetic analysis was conducted. Karyotyping via G-banding was performed on the three iPSC lines (CL12, CL27, and CL30) at passage 15 to screen for chromosomal aberrations potentially acquired during reprogramming or extended culture.

**Figure 2:**
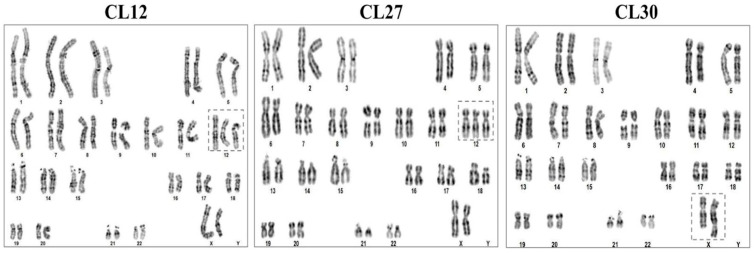
Karyotypic analysis reveals acquired chromosomal abnormalities in iPSC lines obtained from the XP-C patient. Representative karyograms for iPSC CL12, CL27, and CL30 lines are shown. The analysis identified trisomy of chromosome 12 in lines CL12 and CL27, and a derivative X chromosome in line CL30 (dotted-line boxes).

This cytogenetic analysis revealed that all three lines had acquired distinct chromosomal abnormalities, indicating the emergence of genomic instability during in vitro culture. The line CL12 exhibited trisomy of chromosome 12, identified in 4 of 17 metaphases (~23.5%). The line CL27 displayed the same trisomy 12 aberration but at a higher frequency, observed in 12 of 31 cells (~38.7%), suggesting progressive clonal selection. In contrast, the line CL30 harbored a derivative X chromosome, which was detected in 5 out of 36 analyzed metaphases (~13.9%) (Figure 2). All analyzed cells maintained a 46,XX karyotypic background. The acquisition of trisomy 12 is consistent with recognized culture-adapted mutations in pluripotent stem cells [25].

### 3.4. Whole Genome Sequencing Profiling of Genomic Integrity

**Figure 3:**
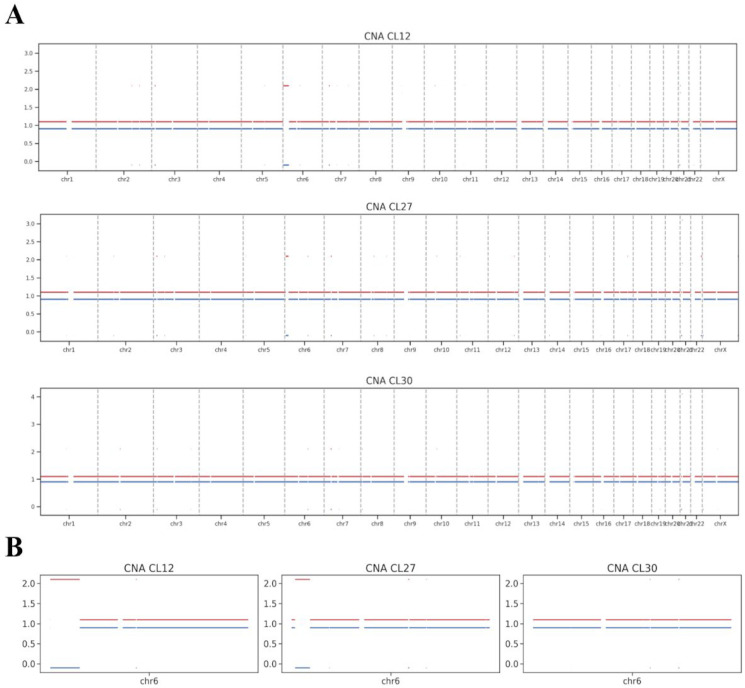
(**A**) Allele-specific copy number analysis of iPSC lines obtained from the XP-C patient. Whole-genome sequencing (WGS) data for lines CL12 (**top**), CL27 (**middle**), and CL30 (**bottom**) are displayed. The plots show allele-specific copy numbers across the genome, with the red and blue lines representing the two parental haplotypes. The *X*-axis indicates chromosomal position, and the *Y*-axis indicates the inferred copy number for each allele. (**B**) Allele-specific copy number analysis of chromosome 6 iPSC lines.

WGS was performed on iPSC lines CL12, CL27, and CL30 at passage 8 to assess chromosomal integrity. Copy number alteration (CNA) analysis of the WGS data did not identify any prominent chromosomal aberrations in these early-passage cells (Figure 3A), including the specific aneuploidies later detected by karyotyping at more advanced passages.

The WGS analysis did, however, reveal subtler structural variations, primarily consisting of copy-neutral loss of heterozygosity (cnLOH) events, which are postulated to arise from mitotic recombination. A notably large cnLOH event was identified on chromosome 6p in lines CL12 and CL27 (Figure 3B). As a copy-neutral alteration, this event is not detectable by standard karyotyping and is unlikely to result in the gross chromosomal abnormalities observed later. Critically, the presence of such cnLOH does not inherently classify the cell line’s genome as abnormal. Lesser copy-neutral structural variations are recognized components of natural genetic variation in human populations.

## 4. Discussion

The successful generation of patient-specific iPSCs has emerged as a transformative approach for modeling human genetic disorders. In this study, we established and characterized novel iPSC lines from a patient with Xeroderma Pigmentosum group C (XP-C), harboring a homozygous loss-of-function mutation in the *XPC* gene (c.1830C>A, p.Tyr610*). While our lines demonstrated robust pluripotency and trilineage differentiation potential, a genomic assessment revealed significant culture-acquired instability, a finding with critical implications for the use of such models, particularly those with underlying DNA repair deficiencies.

The core validation of our iPSC lines confirms their identity and foundational utility. However, the emergence of recurrent chromosomal abnormalities upon extended culture presents a major consideration. The specific anomalies we observed—trisomy 12 in lines CL12 and CL27 and a derivative X chromosome in CL30—are notably non-random. Trisomy 12 is among the most frequently reported culture-adapted mutations in human pluripotent stem cells (hPSCs) [25,26]. Its prevalence is attributed to the conferral of a selective growth advantage, potentially through the dysregulation of cell cycle genes and key pluripotency factors like NANOG located on chromosome 12 [25,27]. Of note, a derivative X chromosome, while less frequently observed, has also been documented in human PSC cultures and may confer a proliferative advantage. Furthermore, other studies have revealed that prolonged passaging induces epigenetic alterations in human ESCs, notably a functional gain of the X chromosome. This gain is attributed to the loss of X-chromosome inactivation in culture-adapted ESCs [27,28]. Thus, our results are consistent with the general model of genomic evolution in hPSC culture, highlighting that this instability is a general property of these cells.

A pivotal finding of our study is the temporal divergence between WGS data from early passage and karyotyping results from passage 15. The absence of large-scale copy number alterations in the early WGS data, contrasted with the later dominance of aneuploid lines, strongly suggests that the major abnormalities were acquired and selected for between these passages. This illustrates a dynamic process of clonal evolution in vitro, where minor, fitter populations overtake the culture over time [29,30]. Interestingly, the WGS data did uncover a substantial copy-neutral loss of heterozygosity (cnLOH) on chromosome 6p in CL12 and CL27. Such events, arising from mitotic recombination, are invisible to conventional karyotyping and represent a subtler, molecular-level form of genomic alteration that can precede overt aneuploidy [31,32].

A critical question emerging from this study is whether the DNA repair deficiency in XP-C cells exacerbates the inherent genomic instability of hPSCs. Although the observed abnormalities are common in hPSC cultures, we posit that the loss of global genome-nucleotide excision repair (GG-NER) fosters a permissive environment for the accelerated accumulation of DNA damage. Given that standard cell culture exposes cells to constant endogenous replication stress and oxidative insult, the absence of the XPC complex—critical for recognizing a broad spectrum of helix-distorting lesions—likely results in an increased burden of stalled replication forks and double-strand breaks, thereby driving genomic instability [33]. The previous hypothesis is supported by studies on other DNA repair-deficiencies, which have demonstrated genomic instability in patient-derived cells [17,18]. Furthermore, our observation stands in contrast to a previous report that described stable XP-specific iPSC lines [19]. This discrepancy could be attributed to several factors, including the specific nature of the *XPC* mutation, differences in reprogramming methods, culture conditions, or the passage number at which stability was assessed. Future studies employing isogenic, XPC-corrected cell lines are required to definitively test this hypothesis and elucidate the specific role of NER in safeguarding genomic stability during reprogramming and pluripotent self-renewal.

## 5. Conclusions

In conclusion, we have established a novel iPSC model from an XP-C patient, which exhibited the expected pluripotent characteristics. However, cytogenetic analysis revealed that prolonged in vitro culture was a predominant factor driving karyotypic evolution in these cells. The acquisition of recurrent, culture-associated abnormalities—specifically trisomy 12 and a derivative X chromosome—directly correlated with extended passaging, highlighting a common vulnerability of pluripotent stem cells under these conditions. While the specific *XPC* mutation did not impede reprogramming or initial validation, its potential role in modulating the rate or spectrum of genomic instability remains a critical question for further investigation. The observed instability, primarily attributable to passaging, underscores the necessity for rigorous and continuous genomic monitoring of all patient-derived iPSC lines, regardless of their genetic background. Future longitudinal studies comparing the clonal dynamics of XP-C lines against wild-type controls across sequential passages will be essential to delineate the specific contribution of the DNA repair defect from the broader pressures of long-term culture.

## Data Availability

The original contributions presented in this study are included in the article/supplementary material. Further inquiries can be directed to the corresponding author(s).

## Supplementary Materials

The following supporting information can be downloaded at: https://www.mdpi.com/article/10.3390/cells14241985/s1, Table S1: Antibodies used for immunocytochemistry/flow cytometry; Table S2: Primers used for amplification reactions and RT-PCR; Figure S1: Flow cytometric characterization of pluripotency in patient-derived iPSC lines; Figure S2: Absence of the reprogramming transgene and Sendai virus; Figure S3: Mycoplasma test results.
