# Supplementary material for: Karyotypic Profiling of Induced Pluripotent Stem Cells Derived from a Xeroderma Pigmentosum Group C Patient
Source: Cells. 2025 Dec 14;14(24):1985. doi: 10.3390/cells14241985 (PMC12732065; doi:10.3390/cells14241985)
Supplement: Supplementary file 1 [file cells-14-01985-s001.zip › cells-4000292-supplementary.pdf]

Supplemental Information

Table S1. Antibodies used for immunocytochemistry/ flow cytometry.

Types of markers	Antibody	Dilution	Company Cat#	RRID
Pluripotency markers	Mouse Anti-TRA-1-60	1:200	Abcam, Cat# ab16288	AB_778563
	Mouse Anti-SSEA4,	1:200	Abcam, Cat# ab16287	AB_778073
	Rabbit Anti-SOX2	1:300	Abcam, Cat# ab97959	AB_2341193
	Rabbit Anti-OCT4	1:300	Abcam, Cat# ab19857	AB_445175
Differentiation markers	Rabbit Anti-NEUROD1	1:300	Affinity Biosciences, Cat# AF0109	AB_2833262
	Rabbit Anti-FOXA2	1:300	Affinity Biosciences, Cat# DF13363	AB_2846382
	Rabbit Anti-Cytokeratin 19	1:300	Affinity Biosciences, Cat# AF0192	AB_2833385
	Rabbit Anti-TUBB3,	1:300	Affinity Biosciences, Cat# AF7000	AB_2846220
	Rabbit Anti-Brachyury	1:300	Affinity Biosciences, Cat# DF13238	AB_2846257
	Rabbit Anti-HAND1	1:300	Affinity Biosciences, Cat# AF0673	AB_2834206
Secondary antibodies	Goat Anti-Rabbit IgG H&L, Alexa Fluor® 594	1:800	Abcam, Cat# ab150080	AB_2650602
	Goat Anti-Rabbit IgG H&L, Alexa Fluor® 647	1:800	Abcam, Cat# ab150079	AB_2722623

	Goat anti-Rabbit IgG (H+L) Highly Cross-Adsorbed Secondary Antibody, Alexa Fluor™ 488	1:800	Invitrogen, Cat# A-11034	AB_2576217
	Goat Anti-Mouse IgG H&L, Alexa Fluor® 488 1:800	1:800	Abcam, Cat# ab150113	AB_2576208
	Goat Anti-Mouse IgM mu chain, Alexa Fluor® 488	1:800	Abcam, Cat# ab150121	AB_2801490

Table S2. Primers used for amplification reactions and RT-PCR.

Type of primer	Target	Size of band	Forward/Reverse primer (5′-3′)
House-keeping genes	*hGAPDH*	150 bp	TTAGCACCCCTGGCCAAGG/CAGGGATGATGTTCTGGAGAGC
Sendai clearance	*SeV*	181 bp	GGATCACTAGGTGATATCGAGC/ACCAGACAAGAGTTTAAGAGATATGTATC
	Klf4	410 bp	TTCCTGCATGCCAGAGGAGCCC/AATGTATCGAAGGTGCTCAA
	KOS	528 bp	ATGCACCGCTACGACGTGAGCGC/ACCTTGACAATCCTGATGTGG
Targeted mutation analysis/sequencing	*XPC*	325	ATGTGGTGGGCATTGACAGT/AGCAACTGCCCCAGCTTTAT
		307	CCTATGTGGTGGGCATTGAC/TGGCTGTGACAATTAAATGAGACA
		343	CCACCAAGCCCATGACCTAT/AGCAACTGCCCCAGCTTTAT
		348	GGGTCCGAGATGTCACACAG/CGCGGCAGTTCATCTTTCA A
		347	TACAAGTACGCCACCAAGCC/TGCCCCAGCTTTATATGGCT
		302	CTGTTACAAGTACGCCACCAA/AACACCCAACATAGTGCTGG

Figure S1. Flow cytometric characterization of pluripotency in patient-derived iPSC lines.

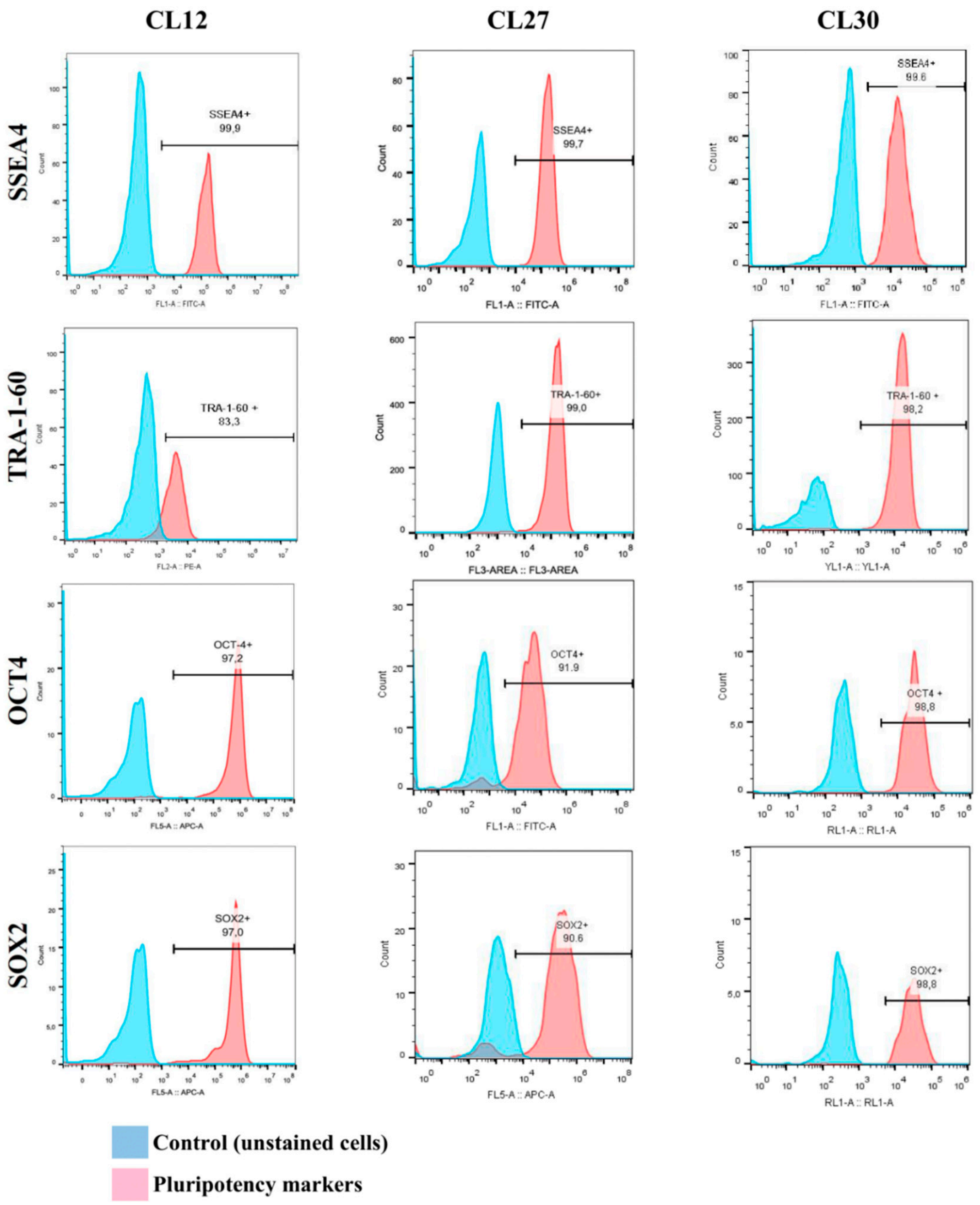

Figure S1. Flow cytometry histograms demonstrating the expression of key pluripotency markers in three established iPSC lines (CL12, CL27, CL30) compared to an unstained control. For each line, the stained cell population (red histogram) is overlaid onto the unstained control (blue histogram).

Figure S2. Absence of the reprogramming transgene and Sendai virus.

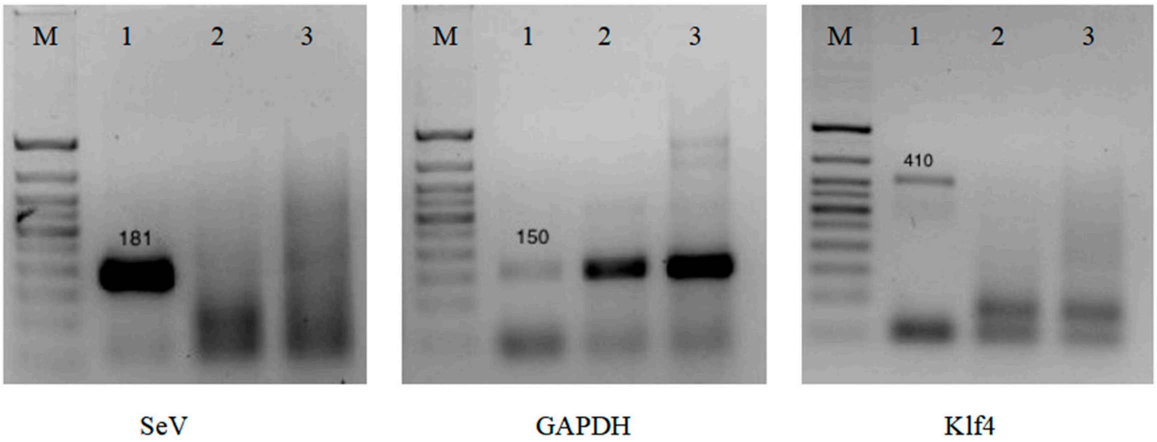

Figure S2. RT-PCR analysis confirming the absence of Sendai virus and reprogramming transgenes in the established iPSCs obtained from one of the lines. Lane M: Quick-Load® Purple +50 bp DNA Ladder (New England Biolabs). Lane 1: Positive control (PBMCs transduced with Sendai vectors). Lane 2: Negative control (PBMCs before transduction). Lane 3: Generated iPSC line. Amplification was performed using primers specific for the Sendai virus genome and the *Klf4* transgene, with *GAPDH* serving as the reference housekeeping gene.

Figure S3. Mycoplasma test results

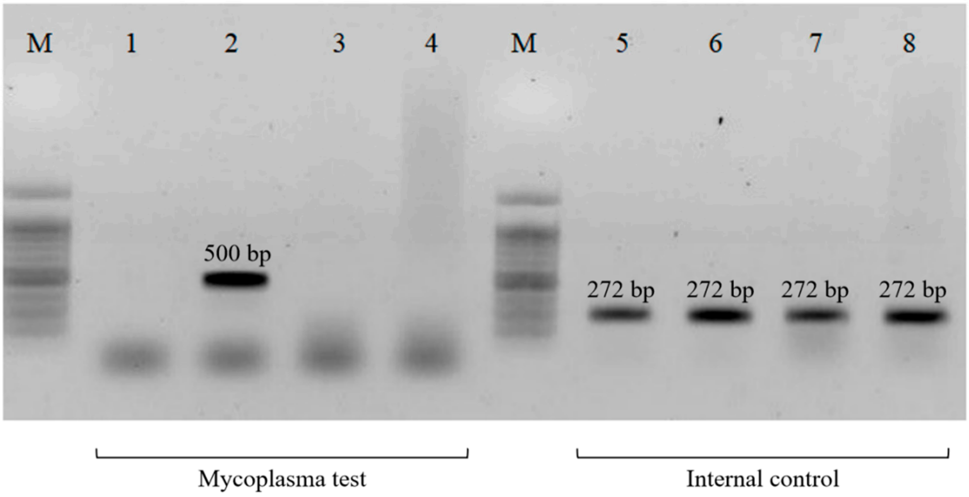

Figure S3. Mycoplasma testing of the iPSCs by PCR obtained from one of the lines. Lanes M, Quick-Load® Purple 100+ bp DNA Ladder. Lanes 1 and 5 are the negative control. Lanes 2 and 6 are the positive control. Lanes 3 and 4 are the iPSC line replicates in mycoplasma test. Lanes 7 and 8 are the iPSC line replicates in internal control. The results confirm the absence of Mycoplasma contamination in the iPSC culture.
